# Total synthesis and biochemical characterization of mirror image barnase†
†Electronic supplementary information (ESI) available: Materials, methods, and detailed experimental procedures. See DOI: 10.1039/c4sc03877k


**DOI:** 10.1039/c4sc03877k

**Published:** 2015-03-23

**Authors:** Alexander A. Vinogradov, Ethan D. Evans, Bradley L. Pentelute

**Affiliations:** a Department of Chemistry , Massachusetts Institute of Technology , 77 Massachusetts Avenue , Cambridge , MA 02139 , USA . Email: blp@mit.edu

## Abstract

In this study we synthesized and characterized mirror image barnase (*B. amyloliquefaciens* ribonuclease). d-Barnase was identical to l-barnase, when analyzed by liquid chromatography and mass-spectrometry. Proteolysis of the mirror image enzyme revealed that in contrast to its native counterpart, d-barnase was completely stable to digestive proteases. In enzymatic assays, d-barnase had the reciprocal chiral specificity and was fully active towards mirror image substrates. Interestingly, d-barnase also hydrolyzed the substrate of the native chirality, albeit 4000 times less efficiently. This effect was further confirmed by digesting a native 112-mer RNA with the enzyme. Additional studies revealed that barnase accommodates a range of substrates with various chiralities, but the prime requirement for guanosine remains. These studies point toward using mirror image enzymes as modern agents in biotechnology.

## Abstract 2

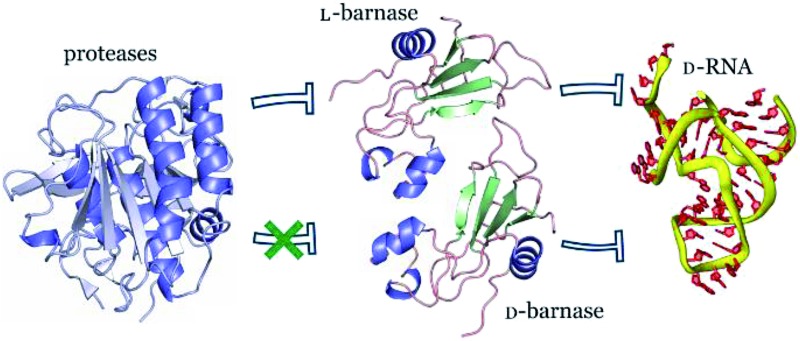
Chemically prepared d-barnase catalyzes hydrolysis of native RNA and appears to be extremely stable to proteolysis.

## Introduction

Mirror image enzymes (MIEs), enantiomers of naturally occurring enzymes, are a promising therapeutically relevant class of biomolecules. These enzymes are thought to be more proteolytically stable and less immunogenic than their native counterparts, while possessing catalytic activity with reciprocal chiral specificity.^1^ Enhanced proteolytic stability and low immunogenicity of mirror image proteins were demonstrated with d-rubredoxin, which was stable to chymotrypsin^2^ and did not elicit an immune response in mice in contrast to its native enantiomer.^3^ The catalytic function of MIEs was studied on the examples of d-HIV-1 protease, which cleaved the d-substrate and not its l-form,^4^ and d-4-oxalocrotonate-tautomerase (4-OT), which acted on the same achiral-substrate as its l-4-OT, but produced the enantiomeric product.^5^ These studies confirmed the reciprocal chiral specificity of MIEs. In a more recent study^6^ the GroEL/ES-assisted folding of mirror image DapA revealed that MIEs may be folded by the native chaperones. Unfortunately, the field of MIEs is still largely unexplored, as only three enzymes were synthesized to date: the mentioned reports represent all published data regarding properties of MIEs.

We undertook this study to systematically investigate properties of an MIE in greater detail. To this end, we synthesized and characterized the enantiomers of *B. amyloliquefaciens* ribonuclease (barnase). Barnase is a potent guanyl-specific,^7^ single strand RNA specific^8^ endonuclease that operates *via* the classical mechanism of RNA hydrolysis, producing a 2′,3′-cyclic phosphate as an intermediate.^9^ The enzyme is more active towards long RNA molecules with the optimum pH at 8.5, but it also hydrolyzes substrates as short as dinucleotides.^10^ We deemed barnase an ideal target for this study due to its structural simplicity (the protein is comprised of a single 110 amino acid residue polypeptide chain with no cysteines^11^), reversible folding–unfolding transition,^12^ and straightforward catalytic activity with a fairly simple readout. Additionally, barnase may be relevant biologically; as bacterial ribonucleases are not inhibited by human ribonuclease inhibitor, barnase exhibits strong cytotoxicity on mammalian cells, and shows promising antitumor activity when conjugated to humanized HER-2 antibody.^13^

## Results and discussion

**Fig. 1:**
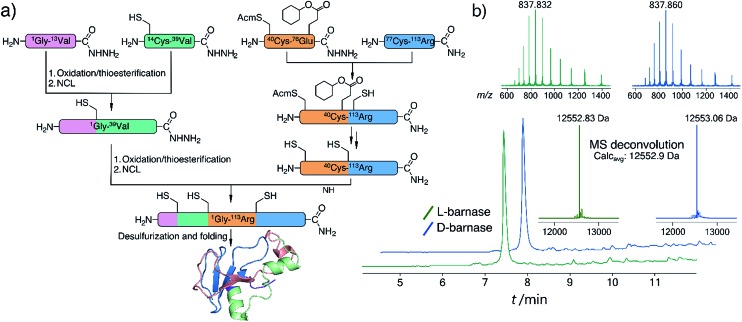
(a) Synthetic strategy for l- and d-barnase. (b) HPLC-MS (TIC) chromatograms for the synthesized proteins with mass spectra insets for the main peaks. Maximum entropy deconvolution spectra of the MS spectra on the top are displayed on the bottom. For both proteins main identified contaminants were +32 Da (Ala → Cys, incomplete desulfurization), and –57 Da (Gly deletion).

To synthesize both enantiomers of barnase we used a previously established strategy with minor revisions (Fig. 1a).^14^ In short, four peptide fragments comprising the protein were rapidly assembled on the fast flow peptide synthesis platform^15^ and purified by RP-HPLC. To increase the yields of Liu's oxidation/native chemical ligation (NCL) protocol^16^ we performed all ligations in two steps, isolating intermediate thioesters by RP-HPLC. Thus, the C-terminal hydrazide H_2_N-[Gly^1^-Val^13^]-CON_2_H_3_ was subject to NaNO_2_ oxidation and 4-mercaptophenylacetic acid (MPAA) transesterification, which afforded the C-terminal thioester of the N-terminal fragment. In the second step the thioester was ligated with H_2_N-[Cys^14^-Val^39^]-CON_2_H_3_ to obtain H_2_N-[Gly^1^-Val^39^]-CON_2_H_3_ with 74% yield over two steps (the one-pot procedure used in the original synthesis of l-barnase yielded 67% of the product). In an analogous manner H_2_N-[Cys^40^(Acm)-Arg^113^]-CONH_2_ was synthesized from H_2_N-[Cys^40^(Acm)-Glu^76^(Cy)]-CON_2_H_3_ and H_2_N-[Cys^77^-Arg^113^]-CONH_2_ with 85% yield over two steps, up from 77% yield obtained originally. After acetamidomethyl and cyclohexyl protecting groups were removed from the C-terminal segment, the final NCL reaction between H_2_N-[Gly^1^-Val^39^]-CON_2_H_3_ and H_2_N-[Cys^40^-Arg^113^]-CONH_2_ afforded full length ^14,40,77^Cys-barnase tri-mutant. This step proceeded rather slowly and inefficiently during the original synthesis (>36 hours to go to completion with 34% yield after purification). To accelerate it, we used twofold excess of the N-terminal fragment and increased the concentrations of both peptides, up to 4.5 mM and 9.0 mM. These changes increased the yield of the reaction up to 65% and allowed to run it overnight. Finally, mild desulfurization^17^ with TCEP, MESNa, and VA-044 radical initiator yielded the desired protein, which was refolded from 6 M Gn·HCl in 50 mM Tris, 100 mM NaCl (pH 7.4) buffer. The improved NCL conditions increased the overall yield of the protein to 19% up from original 12% (calculated from the purified starting fragments). Both synthesized proteins contained a Gly_3_ N-terminal tag to facilitate sortase A-mediated ligation future studies. l- and d-barnase prepared in this way were characterized by HPLC-MS (Fig. 1b) and found identical to each other by liquid chromatography and mass spectrometry. Additionally, synthetic proteins were similar to recombinant barnase, which lacked the Gly_3_ tag: the mass difference in the deconvoluted MS spectrum was 171 Da, consistent with extra Gly_3_ for synthetic variants (ESI 3.1†).

**Fig. 2:**
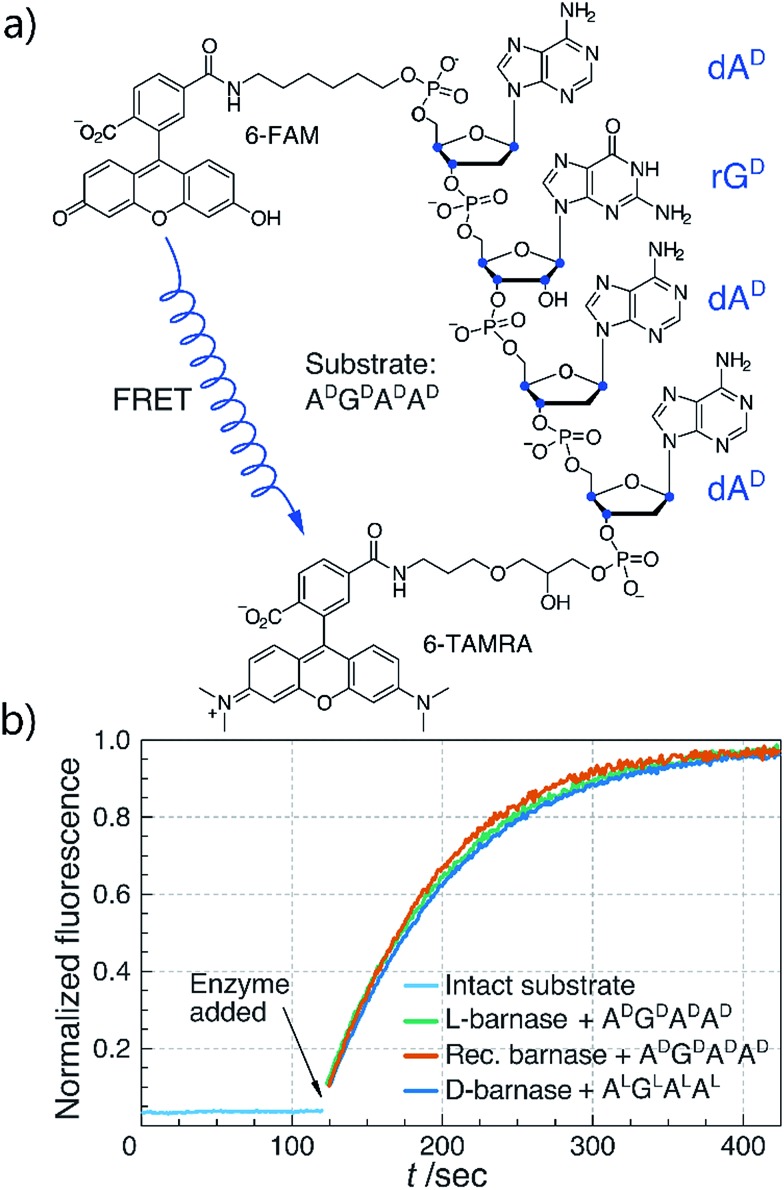
(a) Chemical structure of the A^D^G^D^A^D^A^D^ fluorogenic substrate. Stereogenic centers are highlighted in blue. (b) Biochemical characterization of synthetic l- and d-barnase using the fluorogenic assay. One representative kinetic curve is shown for each enzyme. Barnase concentration was 1.0 nM in all cases.

With both l- and d-barnase proteins in hand we turned to characterizing the catalytic activity of these enzymes. As the RNase activity assay we utilized a modified version of the fast, supersensitive fluorogenic assay developed by Raines and colleagues.^18^ The substrates for the assay are DNA/RNA hybrids with a single cleavage site, which provides for a homogeneous substrate needed to establish kinetic parameters for enzyme catalyzed hydrolysis (Fig. 2a). During the cleavage of the substrate, fluorescence resonance energy transfer between 6-carboxyfluorescein (6-FAM) and 6-carboxytetramethyl-rhodamine (6-TAMRA) fluorophores, installed on the 5′ and 3′ respectively, is perturbed. Thus, the increase in fluorescence of 6-FAM at 515 nm upon excitation at 495 nm can be monitored as a function of time to measure kinetics of the substrate hydrolysis. Enzyme kinetic parameters (primarily, *k*_cat_/*K*_M_) can then be obtained by the non-linear regression of experimental data to the enzyme catalyzed first-order rate equation (ESI 2.2†). In this study we investigated several different tetraoligonucleotide substrates of the common structure 6-FAM-dA^X^-rN^X^-dA^X^-dA^X^-6-TAMRA, henceforth A^X^N^X^A^X^A^X^, where N is a certain nucleotide, and the superscript X annotates the chirality of the sugar (d-sugars constitute native RNA and l-sugars—its enantiomer). In a typical assay, enzyme (100 pM to 100 nM) was added to 50–200 nM substrate in MES buffer (100 mM MES, 100 mM NaCl, pH 6.0), and the fluorescence emission was monitored. In cases where enzyme was unable to hydrolyze the substrate completely in under 60 minutes, an additional aliquot of enzyme was added to promote hydrolysis and measure the final fluorescence of the fully hydrolyzed material.

We first compared the catalytic efficiency of synthetic l-barnase to its recombinant analogue. Because barnase is known as a guanyl-specific endonuclease, we studied the hydrolysis of A^D^G^D^A^D^A^D^. We found that synthetic l-barnase had a *k*_cat_/*K*_M_ value of (1.2 ± 0.1) × 10^7^ M^–1^ s^–1^ (Table 1), in line with the activity of the recombinant enzyme ((1.3 ± 0.4) × 10^7^ M^–1^ s^–1^, Fig. 2b). For d-barnase, we expected the reciprocal catalytic activity (*i.e.*, hydrolysis of the mirror image substrate A^L^G^L^A^L^A^L^). Indeed, d-barnase hydrolyzed this substrate efficiently with *k*_cat_/*K*_M_ = (1.1 ± 0.2) × 10^7^ M^–1^ s^–1^, thus confirming our hypothesis. These data allowed us to conclude that both synthetic enzymes had full catalytic activity.

**Table 1:** Catalytic activities of select RNases towards different fluorogenic substrates. *k*_cat_/*K*_M_ values in M^–1^ s^–1^ ± one standard deviation are displayed

	Recombinant barnase	l-Barnase	d-Barnase	RNase A
A^D^G^D^A^D^A^D^	(1.3 ± 0.4) × 10^7^	(1.2 ± 0.1) × 10^7^	(3.0 ± 0.6) × 10^3^	—^*a*^
A^L^G^L^A^L^A^L^	(3.3 ± 0.3) × 10^3^	(3.2 ± 0.2) × 10^3^	(1.1 ± 0.2) × 10^7^	—^*a*^
A^D^G^L^A^D^A^D^	n.d.^*b*^	(6.9 ± 0.8) × 10^4^	(1.0 ± 0.5) × 10^5^	n.d.^*b*^
A^L^G^D^A^L^A^L^	n.d.^*b*^	(1.7 ± 0.1) × 10^5^	(3.0 ± 0.7) × 10^4^	n.d.^*b*^
A^D^C^D^A^D^A^D^	n.d.^*b*^	—^*a*^	—^*a*^	(4.9 ± 0.3) × 10^7^
A^L^C^L^A^L^A^L^	n.d.^*b*^	—^*a*^	—^*a*^	—^*a*^

^*a*^Hydrolysis was not detected (*k*_cat_/*K*_M_ < 1 M^–1^ s^–1^, upper bound estimation).

^*b*^Not determined.

Next, we sought to study the substrate stereospecificity of the enzymes, *i.e.*, to evaluate the hydrolysis of A^L^G^L^A^L^A^L^ by l-barnase and of A^D^G^D^A^D^A^D^ by d-barnase. Unexpectedly, we found significant remaining activity in both cases: the *k*_cat_/*K*_M_ for l-barnase was (3.2 ± 0.2) × 10^3^ M^–1^ s^–1^, and (3.0 ± 0.6) × 10^3^ M^–1^ s^–1^ for d-barnase. Although these values are ∼4000 times lower than the corresponding ones for the native substrates, *k*_cat_/*K*_M_ of 3 × 10^3^ M^–1^ s^–1^ still represents a fairly potent enzyme^19^ with the rate acceleration of ∼10^10^ over the uncatalyzed RNA hydrolysis.^20^ The similarity of *k*_cat_/*K*_M_ values suggest the observation is not due to an artifact or RNase contamination. However, we performed additional experiments to exclude these possibilities. We used barstar, a well-known barnase-specific inhibitor,^21^ to probe its efficiency in the assays. We found that addition of two equivalents of barstar completely abolished the catalytic activity of l-barnase for both A^D^G^D^A^D^A^D^ and A^L^G^L^A^L^A^L^, confirming that l-barnase is responsible for the cleavage of the substrates. Additionally, recombinant l-barnase, obtained independently from synthetic enzymes, had *k*_cat_/*K*_M_ of (3.3 ± 0.3) × 10^3^ M^–1^ s^–1^ towards A^L^G^L^A^L^A^L^. Finally, a common source of RNase contamination are RNase A family enzymes, which are pyrimidine rather than purine specific,^22^ and thus are not expected to cleave the studied substrates. Accordingly, we did not detect hydrolysis of either A^D^G^D^A^D^A^D^ or A^L^G^L^A^L^A^L^ substrates by RNase A of up to 50 nM. Taken together, these data suggested that barnase may accommodate substrates of the opposite chirality.

To further investigate this phenomenon, we studied the hydrolysis of “mixed chirality” substrates, A^L^G^D^A^L^A^L^ and its enantiomer A^D^G^L^A^D^A^D^, by l- and d-barnase. We found that both substrates were hydrolyzed by the enzymes less efficiently than the native substrates, but significantly faster than tetranucleotides with the fully inverted stereochemistry (Table 1). Thus, d-barnase hydrolyzed A^D^G^L^A^D^A^D^, (the recognition guanosine had the correct chirality, while the rest was inverted) only ∼100 times less efficiently than A^L^G^L^A^L^A^L^ with *k*_cat_/*K*_M_ as high as (1.0 ± 0.5) × 10^5^ M^–1^ s^–1^. The second substrate, A^D^G^L^A^D^A^D^, which had only the guanosine chirality inverted, was hydrolyzed by d-barnase ∼350 times slower than its native substrate. These results were corroborated by the data for l-barnase. At the same time, we could not detect hydrolysis of either A^D^C^D^A^D^A^D^ or its enantiomer, A^L^C^L^A^L^A^L^, by l- or d-barnase. As a positive control for this experiment, we demonstrated the efficient hydrolysis of A^D^C^D^A^D^A^D^ by RNase A, which was consistent with previous reports. Interestingly, we could not detect the cleavage of A^L^C^L^A^L^A^L^ by RNase A.

Collectively, these results confirmed that barnase allows variations in the chirality of its substrates. The chirality of the main recognition nucleoside, guanosine, appears to be more important than the chirality of the rest of the substrate. Moreover, it seems barnase is not simply promiscuous because it did not hydrolyze ACAA substrates, where the key guanosine was replaced by a pyrimidine-based nucleoside. Interestingly, we also found that RNase A did not hydrolyze an enantiomer of its native substrate, which implies that the low substrate stereospecificity is not a universal phenomenon amongst digestive ribonucleases.

**Fig. 3:**
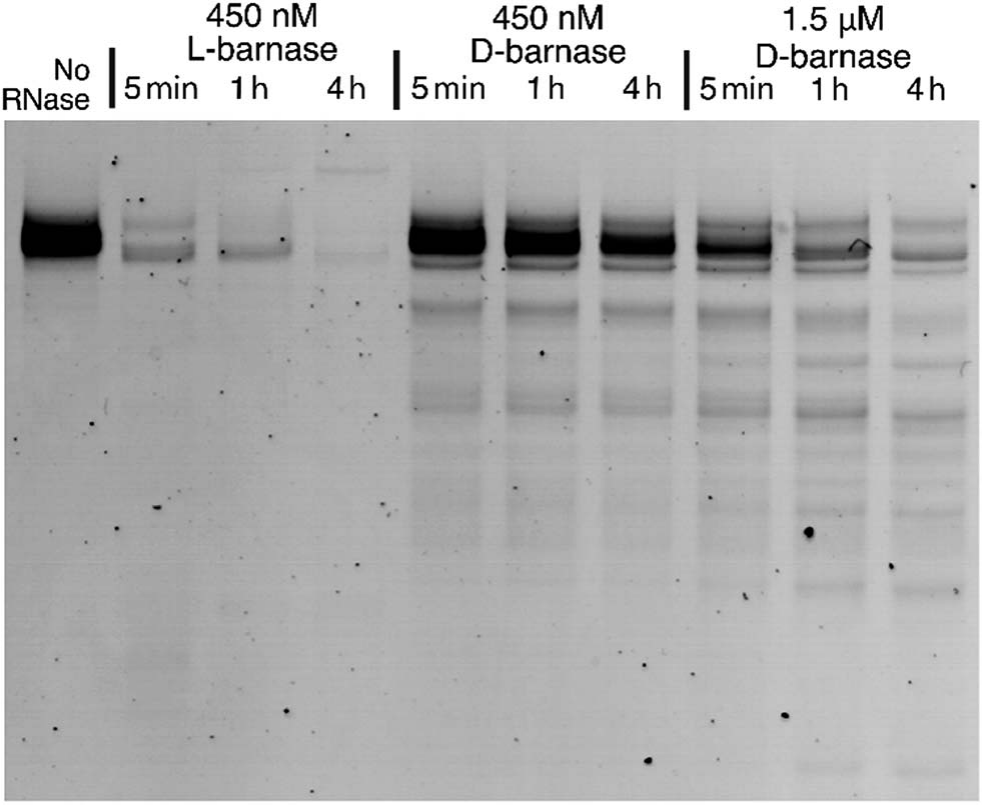
The RNA gel showing the digest of 112 nucleotide-long d-RNA by 450 nM l-barnase, 450 nM d-barnase, and 1.5 μM d-barnase over the course of four hours. The negative control (no RNase added) is shown on the left. The image is digitally modified by inverting the color scheme and adjusting the contrast.

To expand our findings beyond the fluorogenic assay we sought to study the hydrolysis of native RNA by d-barnase. Towards this end, we incubated a 70 μg mL^–1^ solution of a native 112-mer RNA in 10 mM Tris, 50 mM NaCl buffer (pH 7.4) with various concentrations of d-barnase or 450 nM l-barnase for up to 4 hours and analyzed the RNA digest products by performing 10% denaturing PAGE (ESI 3.1†). As demonstrated in Fig. 3, the presence of the low molecular weight bands in cases where d-barnase was added, but not in the negative control lane, indicates that d-barnase cleaved native RNA, albeit slower than l-barnase. The latter observation is evident from digests by 450 nM d-barnase *versus*l-barnase. As such, we confirmed that the results of the fluorogenic assay translate into more complex systems, involving native substrates, and thus, that d-barnase is active towards d-RNA.

In another part of the study we compared the stability of l- and d-barnase towards common digestive proteases *in vitro*. Proteolysis of mirror image proteins was investigated before with metal-bound d-rubredoxin, which was completely stable to chymotrypsin in contrast to its enantiomer.^2^ Although there is evidence for the enhanced proteolytic stability of short, mostly unfolded d-peptides,^23^,^24^ the rubredoxin study remains the only published example of such behavior for folded mirror image proteins. We aimed to study the proteolytic stability of an MIE in greater detail, assaying different proteases and digestion conditions.

As proteases for this study we selected bovine trypsin, α-chymotrypsin, proteinase K, porcine elastase type IV, papain, and *S. griseus* protease (actinase E). These enzymes were chosen for their robust digestive proteolytic activities and a wide range of substrate specificities. Papain represented cysteine superfamily proteases, while other enzymes were serine proteases. Additionally, we wanted to assay enzymes, which are able to recognize and cleave peptide bonds after glycine. Since glycine is achiral, we hypothesized that such proteases may potentially recognize and accommodate glycine residues in mirror image proteins, allowing for the hydrolysis of these substrates. Although glycine-specific digestive proteases are uncommon, both elastase and papain are reported to cleave their substrates after glycine fairly efficiently.^25^,^26^

**Fig. 4:**
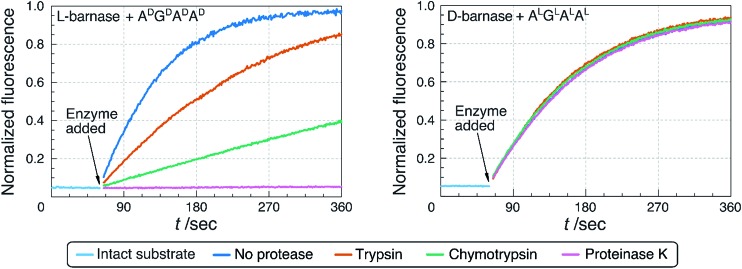
Comparison of the proteolytic stability of l- and d-barnase. Remaining catalytic activity of the enzymes, associated with the extent of the proteolytic digestion, was measured using the fluorogenic assay. Three out of six assayed proteases are not displayed for clarity. Digestion of 1.0 nM l-barnase with different proteases was assayed using A^D^G^D^A^D^A^D^ as a substrate (data shown on the left). Digestion of 0.8 nM d-barnase with different proteases was assayed using A^L^G^L^A^L^A^L^ as a substrate (on the right).

First, we performed the non-denaturing, in-solution digestion of l- and d-barnase by the selected proteases. Proteins were incubated in appropriate buffers (ESI 3.2†) at 37 °C for up to 19 hours with a 15 : 1 ratio of barnase to protease. The extent of the digestion was determined by HPLC-MS analysis (ESI 3.2.1†), and by measuring the remaining ribonucleatic activity *via* the fluorogenic assay. As shown in Table 2 and Fig. 4, we found that after 19 hours of digestion l-barnase demonstrated differential stability towards proteases: trypsin-digested barnase had 36% its native activity, while in the case of proteinase K less than 0.2% activity remained. In all six cases l-barnase lost a significant portion of its catalytic activity. In contrast, d-barnase proved completely stable to all assayed proteases; it retained full catalytic activity, and no digestion products could be detected by HPLC-MS.

**Table 2:** Remaining RNase catalytic activity of l- and d-barnase after the proteolytic digestion with select proteases. Values for the remaining barnase activity are normalized to the negative control experiment, where no protease was added to the enzyme, and are displayed as the percentage of the full ribonuclease activity ± one standard deviation

Protease	l-Barnase	d-Barnase
No protease	100.0 ± 6.4	100.0 ± 5.1
Trypsin	36.7 ± 2.0	108.0 ± 8.9
Chymotrypsin	9.2 ± 0.5	103.1 ± 5.3
Proteinase K	0.2 ± <0.1	99.8 ± 8.1
Elastase	0.6 ± <0.1	103.5 ± 3.3
Papain	0.8 ± <0.1	96.6 ± 4.3
Actinase E	0.3 ± <0.1	101.4 ± 7.3

Additionally, we performed a more forcing in-solution denaturing digestion of l- and d-barnase using the most potent protease, proteinase K. To denature the protein, barnase was incubated in 6 M Gn·HCl, 50 mM Tris buffer (pH 7.4) at 95 °C for 20 minutes, and then digested with proteinase K (barnase : protease = 2 : 1) in 2 M Gn·HCl at 37 °C. We found that d-barnase was completely stable to proteolysis even under such forcing conditions, in stark contrast to l-barnase, which was digested completely (ESI 3.2.2†). Finally, we attempted to digest d-barnase with proteinase K by increasing the digestion time. Using HPLC-MS analysis we did not detect any digestion products after 168 hours (1 week) of incubating d-barnase with proteinase K. The digest was indistinguishable from a negative control experiment, where no protease was added to the enzyme (ESI 3.2.3†).

## Conclusions

In summary, we successfully synthesized and characterized mirror image barnase. We found that the enzyme was fully active towards mirror image RNA model substrates and was somewhat promiscuous to its substrate chirality. After the systematic investigation of this phenomenon we used mirror image barnase to demonstrate the cleavage of the native RNA by a mirror image enzyme. Separately, we found that d-barnase appears to be extremely proteolytically stable. Our experiments revealed that neither cysteine nor serine superfamily proteases are able to cleave it even under forcing conditions. Contrary to our initial hypothesis d-barnase was completely stable towards proteases that are able to cleave peptide bonds after achiral glycine. In short, we were unable to find proteases and/or reaction conditions which would lead to the digestion of d-barnase.

The results of this study pose a number of questions. First, it is unclear how barnase recognizes and cleaves substrates of the opposite chirality. The enzyme is known to have several subsites, which facilitate the substrate binding and its proper orientation for catalysis.^10^ Our data are consistent with this model, as we observed a range of *k*_cat_/*K*_M_ values by only changing the chirality of AGAA tetranucleotide. It is conceivable that substrates of the mixed chirality, *e.g.* A^L^G^D^A^L^A^L^, occupy only certain subsites in the enzyme, *e.g.* the guanosine binding subsite in this case, and thus the catalysis may still proceed. It is also unclear whether this enzymatic activity is merely spontaneous or was subject to the evolutionary selection at some point. At this time we are unaware of any practical implications of such catalysis: to the best of our knowledge, RNA of l-configuration is unknown in nature.

Nevertheless, our study suggests that at least in some cases enzymes may utilize substrates of the opposite chirality. The mirror image form of such an enzyme will then act on the same targets as its native counterpart. Although decreased catalytic efficiency is expected, the enzyme may still achieve a notable rate acceleration. This property of MIEs can be highly desirable from the biotechnology standpoint for, as we confirmed in the case of barnase, MIEs can be extraordinarily resistant to proteolysis and at the same time carry the native biological function. Importantly, this effect may manifest itself without protein engineering and/or evolution of the enzyme. As we also found with the example of RNase A, this effect by no means is universal, and more investigations would be needed to establish the generality of our findings.

## Supplementary Material

Supplementary information
