# Supplementary material for: Total synthesis and biochemical characterization of mirror image barnase
Source: Chem Sci. 2015 Mar 23;6(5):2997–3002. doi: 10.1039/c4sc03877k (PMC5729450; doi:10.1039/c4sc03877k)
Supplement: Supplementary file 1 [file SC-006-C4SC03877K-s001.pdf]

Electronic Supplementary Material (ESI) for Chemical Science.
This journal is © The Royal Society of Chemistry 2015

Mirror image barnase (Supporting Information)

Alexander A. Vinogradov, Ethan D. Evans, and Bradley L. Pentelute

Department of Chemistry, Massachusetts Institute of Technology, 18-596, 77 Massachusetts Avenue, Cambridge, Massachusetts, 02139, United States.

1. Materials 2
2. Methods 2
 2.1. HPLC-MS 2
 2.2. Fluorogenic RNase activity assay 2
 2.3. Protein expression and purification 3
3. Experimental data 4
 3.1. Native RNA digestion by D-barnase 4
 3.2. Proteolysis experiments 5
  3.2.1. Non-denaturing in-solution digestion 5
  3.2.2. Denaturing in-solution digestion 7
  3.2.3. Digestion of D-barnase with proteinase K 8

1. Materials

All reagents for protein synthesis were as described in Mong, Vinogradov *et al.* *ChemBioChem*. 2014, **15**, 721-733. 2-(*N*-morpholino)-ethanesulfonic acid (MES) monohydrate, guanidine hydrochloride (Gn·HCl), sodium hydroxide, hydrochloric acid, Tris base, Tris acid, LB broth, and RNase-free water were from VWR International, Pennsylvania. HPLC-purified tetraoligonucleotides for fluorogenic assays were purchased from ChemGenes, Massachusetts. RNase A was from Qiagen. All other reagents were from Sigma Aldrich and Life Technologies. All reagents were reagent grade and used as received.

2. Methods

2.1. HPLC-MS

All proteins and reaction mixtures were analyzed on an Agilent 6520 Accurate Mass Q-TOF LC-MS using an Agilent Zorbax 300SB C3 column (300 Å, 5 µm, 2.1 x 150 mm). Two methods were used interchangeably. Both methods were operated at 40 °C and a flow rate of 0.8 mL/min. The first method (1-61) used the following gradient: 1% acetonitrile with 0.1% formic acid added (FA, solvent B) in water with 0.1% FA (solvent A) for 2 min, 1-61% B in A ramping linearly over 11 min, 61% B in A for 1 minute. The second method (5-65) used the following gradient: 5% B in A for 2 min, 5-65% B in A ramping linearly over 11 min, 65% B in A for 1 minute. Unless noted, all chromatograms shown in this work are plots of total ion current (TIC) versus time. The data were analyzed using Agilent MassHunter Qualitative analysis software. All MS deconvolution spectra were obtained using the maximum entropy algorithm.

2.2. Fluorogenic RNase activity assay

The assay buffer (100 mM MES, 100 mM NaCl, pH 6.00) was prepared in RNase-free water. Prior to the experiments oligonucleotides were dissolved in the assay buffer, and enzymes were buffer exchanged into the assay buffer. 50–200 nM oligonucleotides and 100 pM to 100 nM enzymes were used to perform the experiments. In a typical experiment, the fluorescence of the substrate solution at 515 nm upon excitation at 495 nm was monitored for 60-300 seconds to measure the starting fluorescence, I_0, and to ensure no background cleavage took place prior to the addition of enzymes. Then, an aliquot of enzyme was added to the substrate and the solution was rapidly mixed. The increase in fluorescence was then monitored for 120–1600 seconds. In cases where the hydrolysis was not complete, an additional aliquot of the appropriate enzyme was added to measure the final fluorescence of the fully digested substrate.

Substrate concentrations in these assays were much lower than K_M values for the enzymes. For example, barnase has K_M = 36.3 µM towards CGAC tetraoligonucleotide (A. Day, D. Parsonage, *et al. Biochemistry*, 1992, **31**, 6390-6395). Thus, k_{cat}/K_M values can be obtained directly from the first order rate equation without resorting to Michaelis-Menten kinetics. The rate equations in this case can be expressed as such:

$$I = I_f - (I_f - I_0)e^{-\frac{k_{cat}}{K_M}[E]t} \quad (1), \quad and$$

$$I = I_0 + (I_f - I_0)\frac{k_{cat}}{K_M}[E]t \quad (2),$$

where I is the fluorescence at time t, I_0 is fluorescence of the intact substrate, I_f is the fluorescence of the hydrolyzed substrate, $[E]$ is the total enzyme concentration, k_{cat}, K_M are steady state enzyme kinetic parameters. The values of I_f were obtained by non-linear least squares regression of (1) with data collected with the addition of sufficient enzyme to hydrolyze all the substrate. The values of k_{cat}/K_M were obtained by either least squares linear (using eq. (2)) or non-linear regression (using eq. (1)) depending on the efficiency and concentration of the enzymes towards particular substrates. All experiments were performed at least in triplicate.

2.3. Protein expression and purification

Barnase was expressed from pMT1002 (Addgene plasmid 8621) in *E. Coli* strain XL-1 Blue (A. Okorokov, R. Hartley *et al. Protein Express. and Purif.* 1994, **5**, 547-552). 1 mL of 6-h culture of *E. Coli* carrying the plasmid grown in LB medium with ampicillin (100 µg/mL), was diluted 1 : 1000 into the same medium and grown overnight at 28 °C, shaking at 220 rpm. Then, (the culture had a density of ~ OD_{600} = 0.6) a further 500 mL of hot (85 °C) LB medium was poured into the cell culture, and the shaker was incubated at 42 °C for another 30 minutes. After that, the temperature was adjusted to 37 °C and cells were cultured for another 16 hours. Acetic acid was added to the culture until the final pH of 4.3, and the cells were centrifuged. The pellet was discarded and 7.5 mL SP-Sepharose resin was added to the supernatant. After stirring for 2 hours at 4 °C the resin was allowed to settle, and the supernatant was loaded on the 5 mL HiTrap Capto S column (GE Healthcare, UK). Pure barnase was obtained by cation exchange chromatography and was buffer exchanged into appropriate buffers using a HiPrep 26/10 Desalting column. The protein purity was assessed by HPLC-MS and PAGE.

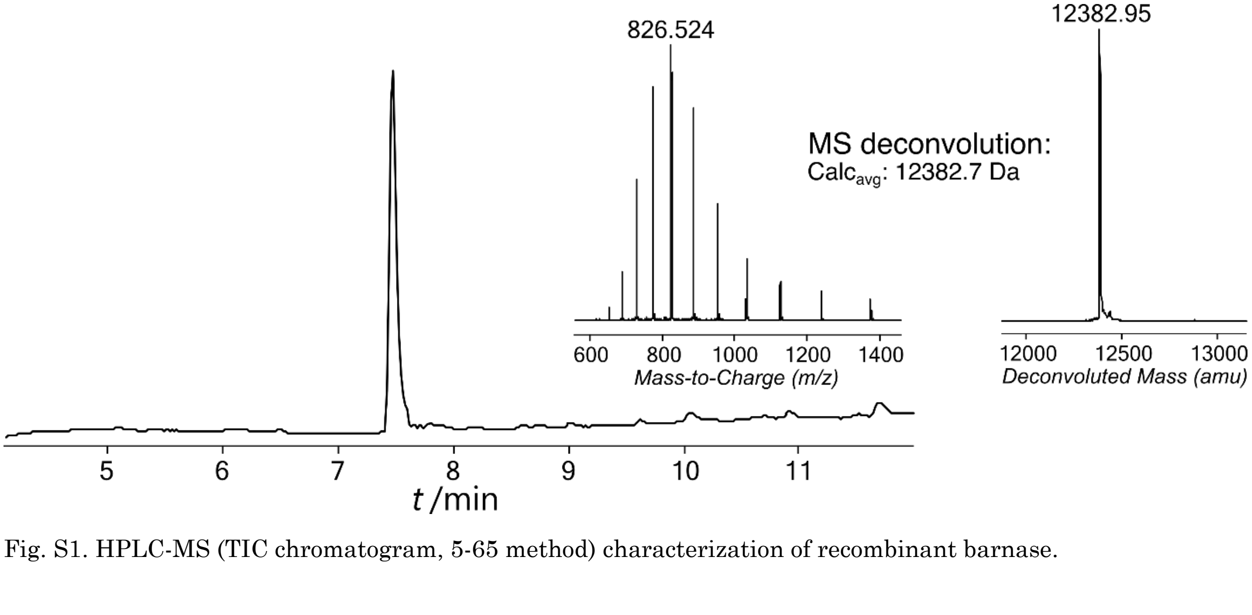

Fig. S1. HPLC-MS (TIC chromatogram, 5-65 method) characterization of recombinant barnase.

The barstar gene was cloned from pMT1002 into a pET-SUMO vector and expressed in *E. Coli* strain BL21 (DE3). The cells were cultured in LB medium with 30 µg/mL kanamycin at 30 °C until OD_{600} was 0.6, and then induced with 0.4 mM IPTG and grown at 37 °C overnight. Cells were centrifuged and pellets resuspended in 100 mL of 50 mM Tris, 150 mM NaCl, pH 7.5 buffer containing 200 mg lysozyme, 4 mg Roche DNase I, and 2 tablets of Roche protease inhibitor cocktail. The cells were lysed using sonication and the suspension was centrifuged further. The lysate was loaded onto 5mL GE HisTrap FF crude Ni-NTA column pre-equilibrated with binding buffer (20 mM Tris, 150 mM NaCl, pH 8.5). The column was washed with 50 mL binding buffer and then 50 mL binding buffer containing 40 mM imidazole. Barstar was eluted with 50 mL 500 mM imidazole in the binding buffer and buffer exchanged to remove imidazole using a HiPrep 26/10 Desalting column (GE Healthcare, UK).

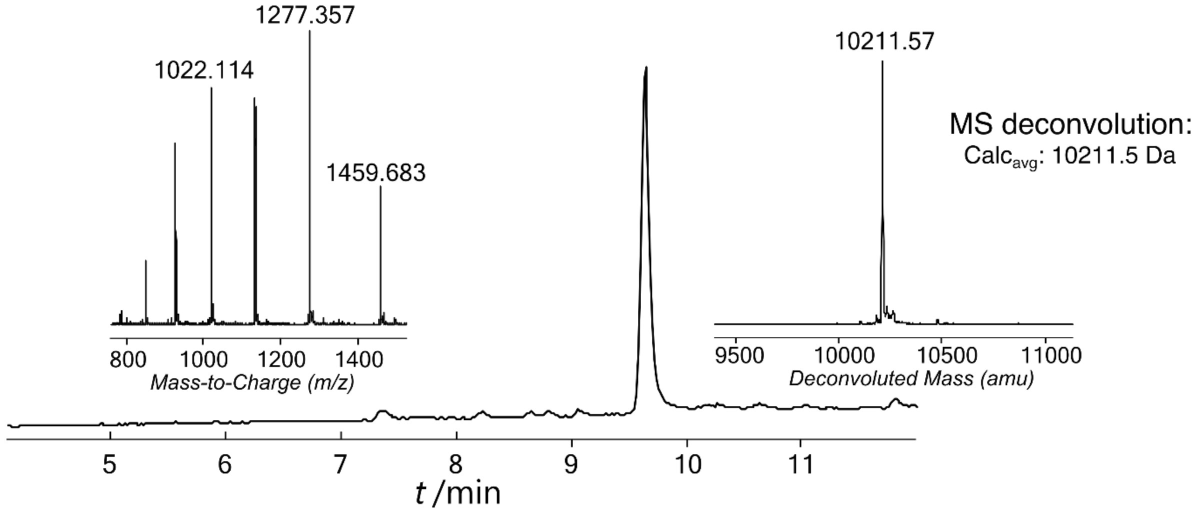

Fig. S2. HPLC-MS (TIC chromatogram, 5-65 method) characterization of recombinant barstar.

Concentrations of proteins were determined spectrophotometrically at 280 nm using appropriate extinction coefficient values and confirmed by HPLC-MS using the standard calibration curves.

3. Experimental data

3.1. Native RNA digestion by D-barnase

The 112mer RNA (5' - GGG ACA AUU ACU AUU UAC AAU UAC AAU GGG UUG CUU CAC GAA CGG GUU GUU GUA CGA GUC CAA GGC GUG CGG CGG UAG CUU AGG CCA CCA UCA CCA UCA CCA CCG GCU AUA G - 3') was prepared via T7 run off transcription from a double stranded DNA template (5' – TAA TAC GAC TCA CTA TAG GGA CAA TTA CTA TTT ACA ATT ACA ATG GGT TGC TTC ACG AAC GGG TTG TTG TAC GAG TCC AAG GCG TGC GGC GGT AGC TTA GGC CAC CAT CAC CAT CAC CAC CGG CTA TAG - 3') and purified by 8% denaturing polyacrylamide gel electrophoresis. Following ethanol precipitation, the pellet was dissolved in 10 mM tris, 50 mM NaCl, pH 7.4 buffer and the concentration was determined spectrophotometrically at 260 nm.

RNA cleavage reactions were initiated by addition of barnase (L- or D-) to a solution of RNA in 10 mM tris, 50 mM NaCl, pH 7.4 buffer, and left at room temperature. A control RNA solution, lacking barnase, was similarly prepared and left at room temperature. The reactions were quenched at the desired time points by the addition of 4 µl of reaction solution to 16 µl of denaturing gel loading dye (8 M urea, 20 mM EDTA, 0.5% bromophenol blue, 5mM tris pH 7.5) and rapidly frozen on liquid nitrogen. Immediately prior to gel analysis, the aliquots were heated to 95 °C for 5 minutes and then run on a 10% denaturing polyacrylamide gel, strained with ethidium bromide and visualized on the Red Gel Imager (ProteinSimple, USA).

3.2. Proteolysis experiments

3.2.1. Non-denaturing in-solution digestion

The experiments were performed in the following buffers:

- For trypsin, chymotrypsin and proteinase K experiments: 50 mM Tris, 100 mM NaCl, 5 mM $CaCl_2$ buffer (pH 7.4);
- For elastase and actinase E experiments: 50 mM Tris, 50 mM NaCl, buffer (pH 8.0);
- For papain experiments: 50 mM NaH_2PO_4, 10 mM EDTA, 10 mM L-cysteine buffer (pH 6.3);

Proteins were incubated in appropriate buffers at 37 °C at a 15 : 1 ratio of barnase to protease. HPLC-MS analysis was performed after 19 hours of incubation time. The reactions were quenched by adding excess 50% acetonitrile/50% water with 0.1% TFA added. The results of the analysis are summarized in Figures S3 and S4.

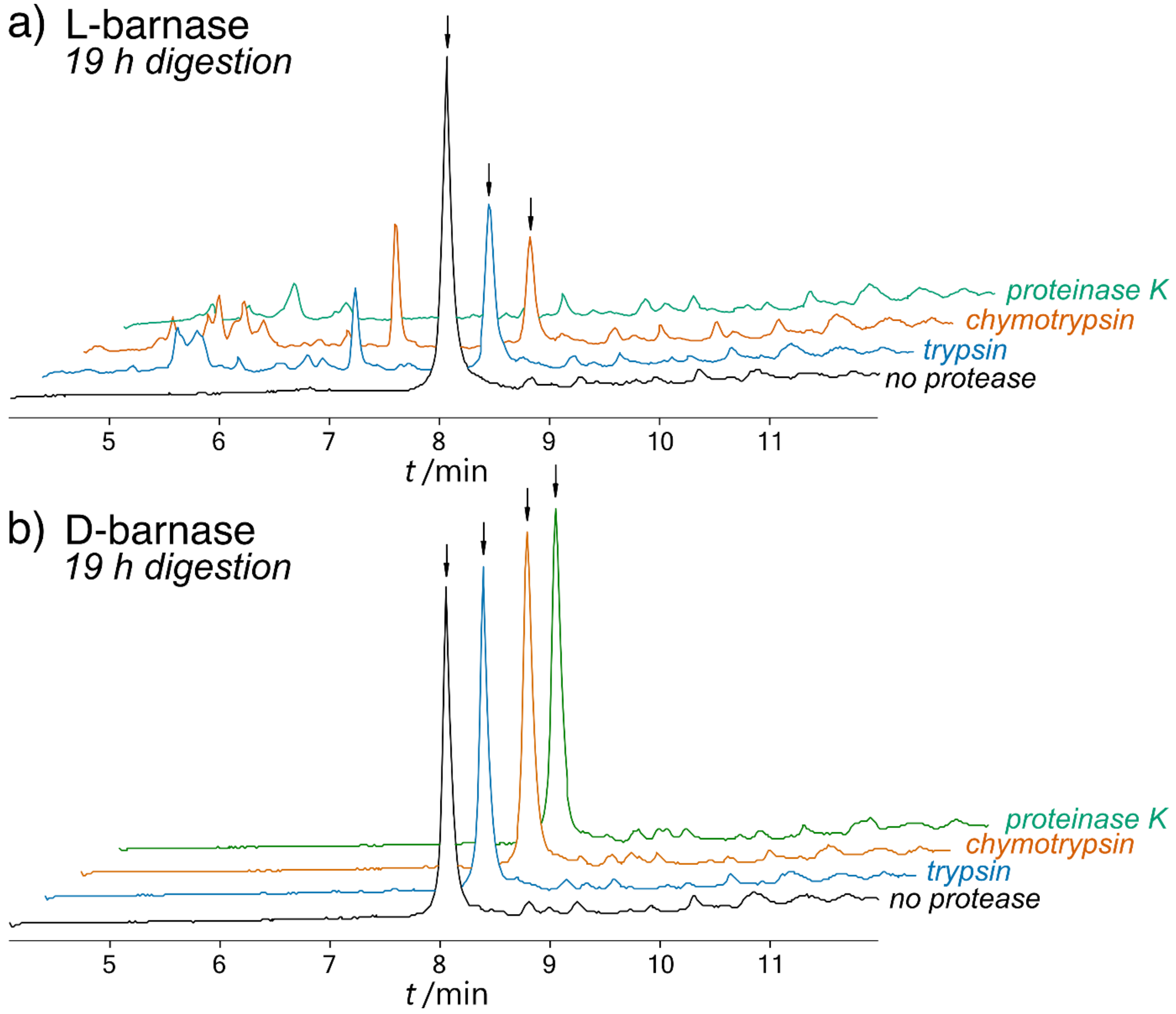

Figure S3. Comparison of stabilities of L- and D-barnase towards trypsin, chymotrypsin and proteinase K under non-denaturing digestion conditions using HPLC-MS (TIC chromatograms are displayed, 1-61 chromatography method was used). Barnase peaks are labelled with arrows.

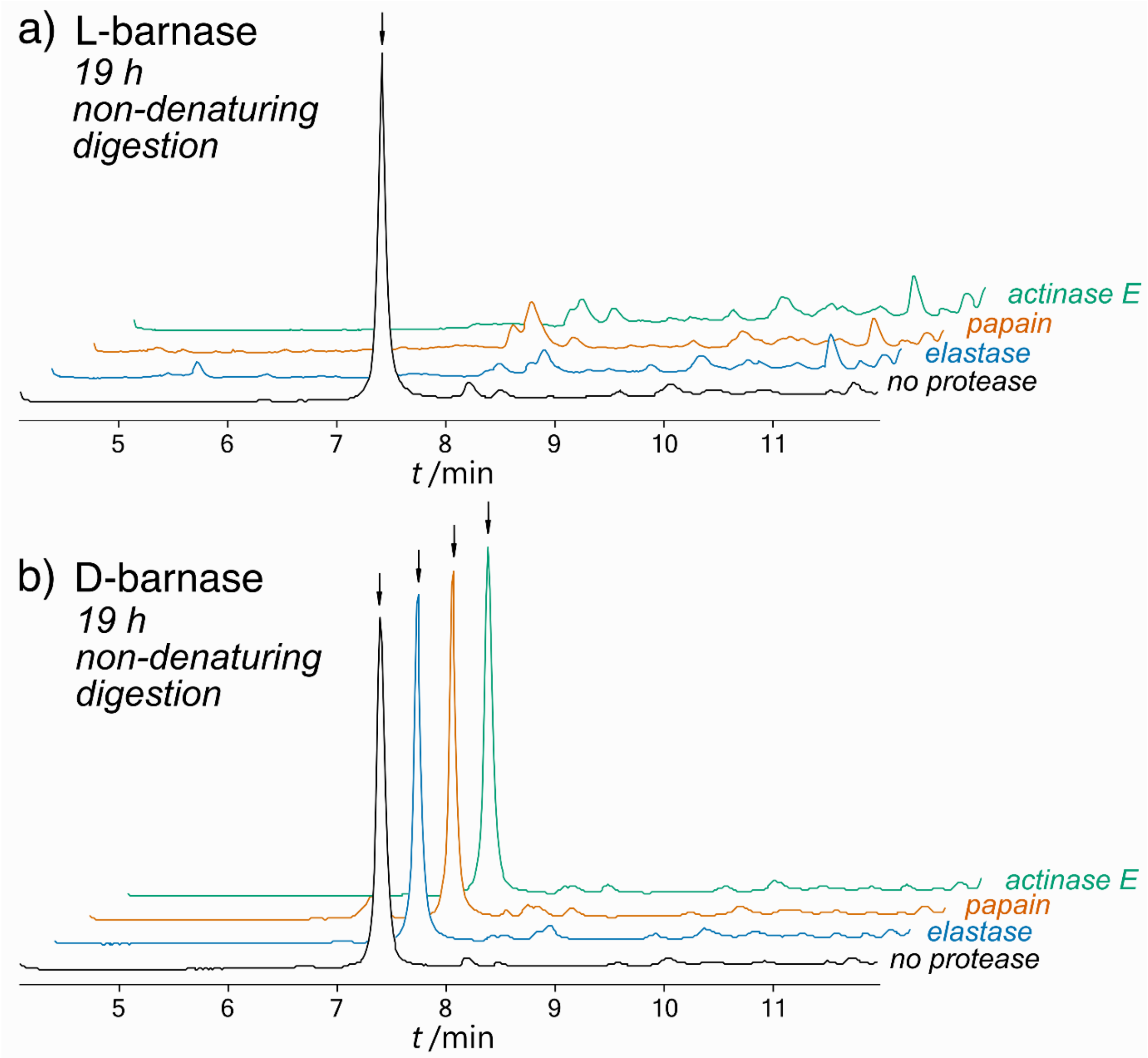

Figure S4. Comparison of stabilities of L- and D-barnase towards elastase, papain and actinase E under non-denaturing digestion conditions using HPLC-MS (TIC chromatograms are displayed, 5-65 chromatography method was used). Barnase peaks are labelled with arrows.

3.2.2. Denaturing in-solution digestion

The experiment was performed as described in the manuscript. HPLC-MS analysis was performed after 4 hours of incubation time to compare L- and D-barnase. Additionally, D-barnase was analyzed after 19 hours of incubation with proteinase K, and was also found to be completely stable. The reactions were quenched by adding excess 50% acetonitrile/50% water with 0.1% TFA added. The results of the analysis are summarized in Figure S5.

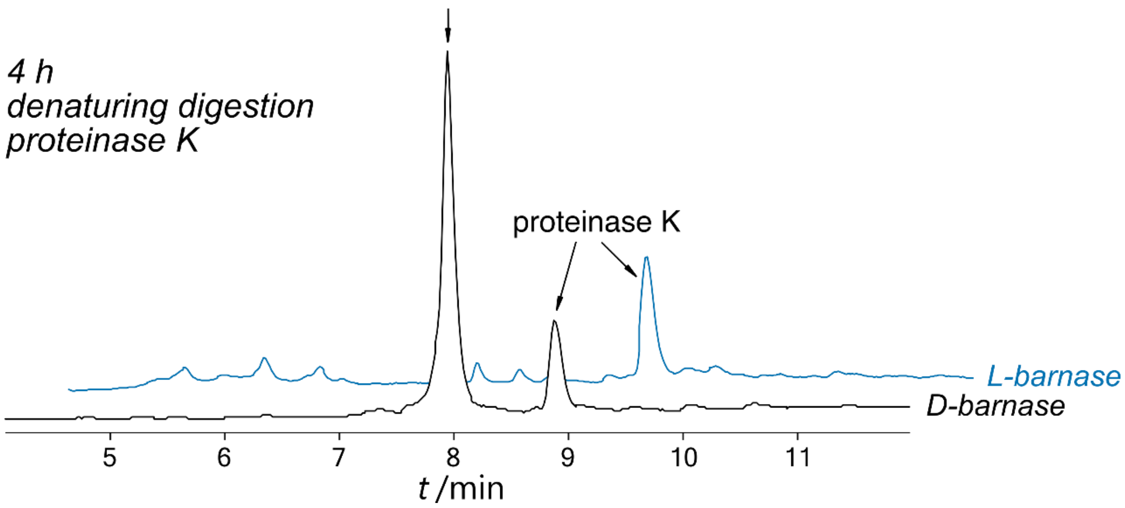

Figure S5. Comparison of stabilities of L- and D-barnase towards proteinase K under denaturing digestion conditions using HPLC-MS (TIC chromatograms are displayed). The barnase peak is labelled with an arrow. In the case of L-barnase no barnase peak was identified after 4 hours.

3.2.3. Digestion of D-barnase with proteinase K

The week-long non-denaturing in-solution digestion of D-barnase by proteinase K was performed under the following conditions. The digestion mixture containing 1.28 mg/mL D-barnase and 0.26 mg/mL proteinase K in 50 mM Tris, 100 mM NaCl, 5 mM $CaCl_2$ buffer (pH 7.5) in a total volume of 25 µL was incubated at 37 °C for one week. A sample for HPLC-MS analysis (1 µL) was taken every 48 hours, and additional proteinase K (1 µL of 1.28 mg/mL stock in the same buffer) was added to the digestion mixture at the same time. As a negative control experiment, D-barnase was incubated under identical conditions free of proteinase K. The reactions were quenched by adding excess 50% acetonitrile/50% water with 0.1% TFA added. The HPLC-MS data of the digestion endpoint are summarized in Figure S6.

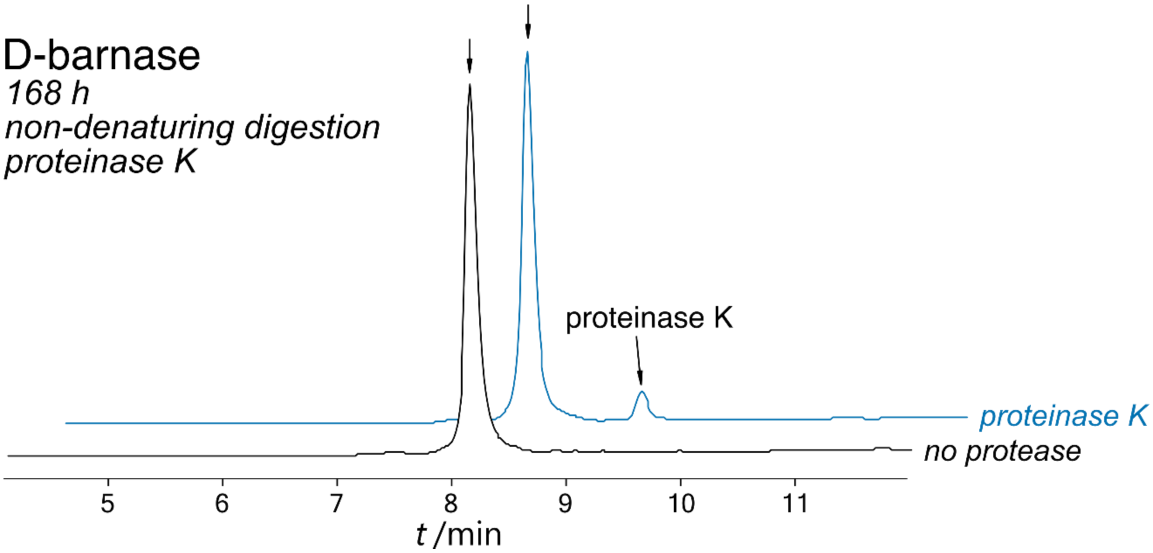

Figure S6. Digestion of D-barnase by proteinase K under non-denaturing digestion conditions for 168 hours. HPLC-MS TIC chromatograms are displayed. Barnase peaks are labelled with arrows.
